# Round the Hospitals

**Published:** 1919-11-22

**Authors:** 


### ROUND THE HOSPITALS.

At the Investiture held at Buckingham Palace on November 13, the King personally conferred decorations on members of the Nursing Services as follows: Royal Red Cross, First Class—Miss Ida Pooley, Q.A.I.M.N.S.; Miss Mary Blair, Q.A.I.M.N.S.R.; Matron Rose Creal, Australian A.N.S. Royal Red Cross, Second Class—Mrs. Anna Gaze, Q.A.I.M.N.S.; Miss Ida Bull, Miss Dorothea Grant, Miss Jane Hawkins, and Miss Winifred Linton, Q.A.I.M.N.S.R.; Miss Nora Dillon, St. John's Brigade; Miss Ursula Hall and Miss Hilda Martin, V.A.D. The King conferred the Military Medal for bravery in the field on Miss Christina McLean, Q.A.I.M.N.S.R.,

**ROUND THE HOSPITALS**—(*continued*).

and also on Miss Mary Blair, to whom he had already presented the Royal Red Cross, First Class. We believe this to be the first occasion on which a nursing sister has received this double decoration at the hands of the King.

The King held another Investiture on November 15, and personally conferred the following decorations on members of the Nursing Services:—Royal Red Cross, First Class.—Miss Castalia Gibb, Q.A.I.M.N.S.; Miss Margaret Gordon and Miss Mary O'Neill, Q.A.I.M.N.S.(R.).—Royal Red Cross, Second Class.—Miss Susan Clarke, Miss Eleanor Davies, Miss Euphemia Kay, and Miss Evelyn Moore, Q.A.I.M.N.S.(R.); Miss Mary Brownlees, Miss Mathilde Bull, Miss Ruby Gilchrist, and Miss Catherine Pierce, T.F.N.S.; Miss Harriet Fraser-MacDonald, B.R.C.S.; Sister Mary Bartlett, Sister Elizabeth Dalzell, Sister Jessie Gibson, and Sister Evelyn Nobbs, Australian A.N.S. Also Miss Margaret Berry, Miss Marjory Heaton-Ellis, Mrs. Jean Henderson, Miss Alexandra Moss, Miss Hilda Sandys, Mrs. Amy Scrimshaw, and Miss Mary Smith, V.A.D. His Majesty conferred the Military Medal on Miss Winifred Brampton, V.A.D.

The visit of the Prince of Wales to Washington was made memorable from a nursing point of view by the presence of the American Army nursing sisters at the Investiture which he held on November 13 in the drawing-room of his temporary residence, Mr. Percy Belmont's house. The Prince was received by a large number of naval and military representatives of the different embassies, and conferred decorations on numerous American officers and members of the American Flying Corps. One Army nursing sister received from his hands the Order of the Royal Red Cross, First Class, and seven the Associateship of the Order, which binds in one fellowship nursing sisters from all parts of the Empire and many beyond its bounds.

The Lord Mayor has handed to the French Ambassador the sum of £75,000, the proceeds of the collections made in Great Britain and Canada on "France's Day," towards the work carried on by the British Committee of the French Red Cross. This Committee is now devoting the whole of its energies and resources to the relief of the suffering occasioned by the war in France. The numbing effect of past wretchedness on the energies of the French people is one of the marked features of post-war conditions. To break up this hopeless apathy and awake by timely help some smouldering hope in those who have forgotten how to hope is the work of the Red Cross. It calls for more and more money, and now is the time to contribute it.

We note that the American Red Cross Commission for Great Britain and Ireland is finishing its work and planning to return to America. It has left its mark in this country in more than one direction, and it is not too much to say that generations of infants yet unborn will have cause to bless its generous operations. The London Chapter of the Society, of which Viscountess Harcourt is chairwoman, will maintain the interests of American Red Cross work here for the future. Lady Harcourt has set herself to enrol every American in England, and requests that names may be sent in to her at 80 Lombard Street, E.C. 3.

It is impossible to resist a smile over the "Bill to Provide for the Registration of Nurses." All the thorny points have been neatly filed off; every paragraph which might afford occasion for controversy has been eliminated; the outline is barely indicated; the light and shade and colour are all to come. Instead of the careful definition of training provided in other Bills, we have merely such expressions as persons who shall have undergone "the prescribed training," and "shall possess the required experience." The prescribed training is to be "either in an institution approved by the Council in that behalf, or in the service of the Admiralty, the Army Council or the Air Council." There are to be supplementary parts for male nurses, mental nurses, nurses trained in the nursing of sick children and "any other prescribed part."

The really controversial matter, it has always been recognised, in a Bill for the registration of nurses is the constitution of the Council. Here again, Dr. Addison has succeeded to admiration in disarming criticism. At its first constitution the Council is to consist of twenty-five persons, two, who are not to be doctors or nurses or persons concerned with the regular direction of nurses, are to be appointed by the Privy Council; five are to be appointed by the Minister of Health after consultation with nursing authorities and experts; and sixteen must be or have been trained nurses, and these are also to be appointed by the Minister of Health after consultation with the Central Committee for the State Registration of Nurses, the College of Nursing, the Royal British Nursing Association, and such other bodies concerned as may desire to be consulted. It was perhaps the only way in which a Registration Bill could secure unanimous support, but it will leave some supporters of the Registration principle feeling a little cold. When the first Council expires, which will be not later than three years after its inception, the sixteen nurses specified above will be elected "in accordance with the prescribed scheme," the nine other persons being appointed as before. Dr. Addison and his advisers have won the unanimous vote of the Commons. They are entitled to the warmest congratulations of the nursing profession and the public.

The appearance of the Register of Nurses put forth by the College of Nursing could not possibly have been more timely. It contains 16,000 names, among which will be found those of prac-

**ROUND THE HOSPITALS**—(*continued*).

tically all the leaders of the profession. What it represents in minute care, research, organisation, voluntary effort, skill and patience, last but not least in money, is hard for any but those most intimately acquainted with the work to appreciate. Our sincere congratulations are offered to the permanent staff, who have laboured ceaselessly for many months past to get this volume of information ready for the press and have succeeded in incorporating even the latest accretions to the membership of the College. All whose names are inscribed on the list should also share in the congratulations. To have their names on this first register is a matter of just pride, for the future of the nursing profession will be built on this foundation.

---

We are very glad to have from the Ministry of Pensions a clear statement that "a nurse who has been discharged or demobilised from any of the Services, and is in receipt of retired pay or gratuity in lieu of retired pay for a disability due to or aggravated by war service, is entitled to assistance from the Ministry of Pensions." Should a nurse be suffering from such a disability and have received neither retired pay nor gratuity, she is entitled to have her claim to retired pay and medical treatment considered. In all cases she should communicate in writing with the Secretary M.S. 2 (Officers) Ministry of Pensions, 14 Great Smith Street, Westminster, S.W. 1. Nurses should forward medical evidence of the present conditions of their disability and some evidence of its being due to their service if possible. If a nurse merely requires advice on a question of retired pay or medical treatment, she is recommended to apply to the Officers' Friend, Ministry of Pensions, Westminster House, Millbank, S.W. 1.

---

The eight nursing sisters discharged by the Scottish Branch of the Red Cross Society from their hospital at Rouen in March 1918, have lodged with the Secretary, a formal request for inquiring into their grievances signed by over seventy representative contributors. Readers will remember the case, which had some painful features. No stigma was thrown on the character or professional competence of those who had been dismissed, but we were not altogether satisfied with the manner in which their plea was presented. We think they are entitled to be heard in their own defence, and trust that the provocative tactics which were employed in their behalf will not be allowed to prejudice their case.

---

Peace has left its mark for good on the County Hospital at York. The large hut which was used as a military ward during the war has been converted into an excellent new women's surgical ward containing twenty beds, together with an adjoining orthopædic and massage department. These were dedicated on November 13 by the Rev. Q. F. Perkins, chaplain of the hospital. The extra nurses needed owing to this enlargement and the shortening of duty hours are accommodated in six new nurses' bedrooms added to the Home, reconstructed from Army huts. The walls are pale grey and the furniture is in white enamel, and the effect is charming.

---

A physician, writing in the *Manchester Despatch* on the high cost of nursing homes, cites an instance which has come to his knowledge "where the united salaries of two nurses, who had to attend to four patients, were £4 2s. a week, whereas the patients were paying in the aggregate £38 a week." Diets, such as oysters and chicken, were charged as extras, together with all dressings, lint, etc. The doctor in question suggests that the patient should adapt a room in his own house by clearing out carpets, curtains, etc.; with an efficient nurse installed he argues that the patient has all that is wanted for recovery at a tithe of the cost. But there must be at least two nurses for a case nursed at home. Their salaries would cost £5 5s. to £6 6s.; their board £1 1s. a week each; besides their laundry, extra maid to attend to their requirements, patient's food, chemist's bill, and extras (which, after all, cost a good deal); total certainly not less than £10 to £12 a week. The case against the nursing home proprietor is not so conclusive as it sounds.

---

The Lewisham Guardians have already shown their sense of the value of good nursing by instituting an eight-hour day in their fine infirmary. They have recently passed the plans for a new nurses' home, for which there is fortunately ample room in the spacious grounds. The new building is estimated to cost close on £42,000, and as soon as the plans are approved by the Ministry of Health the work will be pushed forward. There will be room for nineteen additional nurses, with study and recreation rooms.

---

The prison of St. Gilles in Brussels, where Miss Cavell and Mlle. Petit were confined prior to their execution by the Germans, has been converted into a museum dedicated to their memory. The last objects connected with these brave women, their little personal possessions, clothes and books, have been brought together and form a touching collection. Tablets draped in flags commemorating their heroic death have been placed on the doors of the two cells. On that of Edith Cavell is the inscription: "In Memoriam Edith Cavell, fusillée par les Allemands le 12 Octobre 1915. Elle se sacrifia pour la Croix Rouge. Elle s'immola pour la patrie." Inside are their portraits wreathed with flowers. The spot will be a place of pilgrimage for nurses for many generations to come.

---

They have decided at Leicester to take commodious premises in the London Road to form headquarters for the local Voluntary Aid Detachment. This will form a centre for storing a large mass of equipment, stretchers, etc., and a garage will be

**ROUND THE HOSPITALS**—(*continued*).

built for motor ambulances. There is to be a reading-room, billiard-room, and gymnasium, and organised social evenings, and dances for members form part of the programme. A register of voluntary workers, ready to assist the nurses as required in times of epidemics, will be a most useful adjunct. The idea is for these workers to look after the homes, do simple cooking, and ensure food and firing to the patients in epidemics. The scheme appears to be admirably well thought out, but it is not quite clear how the social side of the work is going to be financed.

The researches into the causes of accidents in factories which have been carried out by Dr. Major Greenwood and Miss Hilda Woods, and lately reported on by them, have an intimate bearing on the causes of failure in nurse-training schools. They elucidate the fact that among large groups of women workers there will always be first, some whose nervous system is so constituted that it cannot stand pressure of work; secondly, some whose physical health leads exceptionally rapidly to a state of fatigue, and is incapable without disturbance of tolerating changes of temperature, excitement, and so forth; thirdly, some whose careless methods defeat all efforts to control them. The wary matron, guided by reports from ward-sisters, usually succeeds in eliminating the last-named category of probationers fairly early in their destructive career, and the first-named departs peaceably on her own initiative after a brief trial of the pressure of work in hospital. But those who are in the middle category are a far more numerous group which numbers many undeniably good nurses. It is among these, the women whose health is affected, perhaps insidiously, by extra fatigue, who re-act unfavourably to extremes of heat and cold and show sympotoms of instability after excitement or painful sights, that many disabled war sisters are to be found. Physical training is found to have an immediate effect for good in establishing a sound physical and mental equilibrium even under adverse conditions, and ought to be introduced among nurses in training.

Dr. Maitland, resident physician at the Brompton Hospital for Consumption has been urging in the *Daily Graphic* the need for an increase in educative work among nurses and health visitors in the matters of tuberculosis. With 94,405 cases of tuberculosis notified in England and Wales last year it becomes a matter of intense importance to the community that the nurses they employ should be able to view suspiciously early symptoms in the period of weakened resistance and to advise resort to a medical man. Dr. Maitland believes that nurses do not fully realise how much scope there is for them in this work. There is a grave shortage of nurses specialising in this way and he considers the very best type of nurse who tends to prefer surgical work ought to feel that here is a career in which she will have ample opportunities. The problem is a big one, affecting this and future generations, and the nurses who devote themselves to its solution, are assisting as pioneers in experimental work of immense interest and value. The Brompton Hospital by its admirable courses of lectures to intern and extern pupils is doing much to educate the nursing profession.

No one can be surprised to hear that in China only about 1 per cent. of the medical and nursing needs are being met. What is astonishing is that Canada and Newfoundland have set out to secure more adequate facilities for training Chinese doctors, dentists, and nurses. The medical and nursing professions in Canada are rallying to aid West China, and a most enthusiastic meeting was held in Toronto in mid-September of governors of the West China Union University to that end. It is a gallant endeavour.

The great shortage of district nurses which is crippling the work of the County Nursing Associations in this country is reflected also overseas. In Melbourne the Central Council of the Victorian Bush Nursing Association is in a very difficult position owing to the shortage of nurses. Six of the thirty centres are without any nurses at all, and the supply of midwives is also very inadequate. Under the circumstances the usual cry is being raised that nurses do not need such long training, and that nursing V.A.D.s who have had a few months' experience in the art of making patients comfortable will "do just as well." Some of the Victorians are particularly annoyed that their State lays down a minimum period of twelve months' training for midwives unless they are already trained nurses. Our English midwives are therefore not eligible to practise in Victoria, and the Bush Nursing Association has to get special permission to employ nurses who hold the certificate of the English C.M.B. as "emergency" nurses.

The nurses of the Redhill Infirmary have awaked to find themselves famous; more than one of the leading daily papers have devoted space to the spirited protest they addressed to their Board on the subject of the accommodation provided for them. Will it be believed that no sitting-room has been provided for their use except one in which meals were being served all day long? The result was that they were driven to take refuge during off-duty hours in their bedrooms—a cheerless prospect indeed as their quarters are at some distance from the Infirmary. Nurse Penny and Nurse Atkinson courageously obtained permision to attend the Board meeting as a deputation and laid their hard lot before the Guardians who, if culpably careless in ignoring for so long the discomforts of their staff, proved highly sympathetic when they found all the nurses had written out their notices and would hand them in if their grievances remained unredressed. The Works Committee have instructions to find more suitable quarters without delay and the nurses are to be provided with cloaks and goloshes. There is even talk of a subscription to buy the nurses a piano.

